# Insecticidal and Repellent Activity of Different Pomegranate Peel Extracts Against Granary Weevil Adults

**DOI:** 10.3390/insects16121222

**Published:** 2025-11-29

**Authors:** Federica Lo Muzio, Onofrio Marco Pistillo, Ilaria D’Isita, Giovanni Iadarola, Antonella Di Palma, Antonio De Cristofaro, Giuseppe Rotundo, Giacinto Salvatore Germinara

**Affiliations:** 1 Department of Agricultural Sciences, Food, Natural Resources and Engineering (DAFNE), University of Foggia, Via Napoli 25, 71122 Foggia, Italy; federica.lomuzio@unifg.it (F.L.M.); marco.pistillo@unifg.it (O.M.P.); ilaria.disita@unifg.it (I.D.); giovanni.iadarola@unifg.it (G.I.); antonella.dipalma@unifg.it (A.D.P.); 2 Department of Agriculture, Food, Environment (Di.A.A.A), University of Molise, Via de Sanctis, 86100 Campobasso, Italy; decrist@unimol.it (A.D.C.); rotundo@unimol.it (G.R.)

**Keywords:** pomegranate, bioassays, storage pests, bioactive compounds, bioinsecticides

## Abstract

The pomegranate (*Punica granatum* L.) fruit peel is an agro-industrial by-product rich in bioactive compounds. In this study, the bioactivity of pomegranate peels (cv. Ako) extracted with acetone, diethyl ether, and *n*-hexane was assessed by evaluating toxic (contact and ingestion), repellent, antifeedant, and nutritional effects towards *Sitophilus granarius* (L.) (Coleoptera, Curculionidae) adults. Contact toxicity assays revealed significant mortality induced by the acetone and *n*-hexane extracts, with 24-h LD_50_ values of 76.93 and 81.14 μg/adult, respectively. In ingestion bioassays, at the highest dose tested (750 μg/disk), the acetone pomegranate peel extract showed a strong feeding deterrence (FDI: 80%), and significantly reduced food consumption (RCR) and relative growth rate (RGR). In filter paper repellency assays, the acetone extract induced positive contact repellency, with PR values ranging from 80% to 30%. GC-MS analysis identified sitosterol, 9,12-octadecadienoic acid, and α-tocopherol as the major constituents of the acetone extract. These results highlight the potential of pomegranate peel as a sustainable source of bioactive compounds for stored-product insect pest management.

## Simple Summary

The bioactivity of pomegranate peel extracts, obtained by different polarity solvents (acetone, diethyl ether, and *n*-hexane), was evaluated on adults of the granary weevil, *Sitophilus granarius* (L.), in toxicity and repellency tests. The acetone extract showed the strongest toxicity, significantly reduced feeding activity, and exhibited high contact repellency. Chemical analysis revealed sitosterol as the main component. These results support the valorization of pomegranate peel as a natural resource for sustainable stored-product pest management.

## 1. Introduction

In the contemporary era, one of the main global priorities is to ensure a sustainable and efficient food system by increasing production and implementing effective loss-reduction strategies, as the population is expected to reach 9 billion by 2050 [1]. Post-harvest pest infestations in storage facilities represent a major threat to the quality and safety of agricultural commodities [2]. Cereals, a staple of human nutrition, are particularly vulnerable to insect attacks, which result in considerable economic, and nutritional losses. It is estimated that annually, between 10–20% of global cereal production is lost due to insect infestations when storage conditions are poorly managed [3,4,5]. Traditionally, stored-grain pest management has relied extensively on contact residual insecticides (e.g., organophosphates, carbamates, and pyrethroids) and fumigants (e.g., phosphine) [6,7]. However, the prolonged and widespread use of these chemicals has raised significant environmental and public health concerns, including the emergence of resistant insect populations, increased pesticide residues in food supplies, and ecological contamination [8]. Consequently, the urgent need for sustainable pest control strategies has driven extensive research towards plant-based formulations, including botanical extracts and essential oils, as alternatives to conventional pesticides, which have been primarily explored under laboratory conditions [9,10,11,12,13]. Among the natural resources being explored, industrial by-products, generated in substantial quantities during food processing [14,15], demonstrated antifungal, antimicrobial, and insecticidal properties [16,17,18,19,20,21]. Pomegranate (*Punica granatum* L., Lythraceae), a fruit native to Central Asia and widely cultivated for its nutritional and health benefits [22], generates substantial amounts of waste during large-scale food processing. However, the fruit peel, accounting for approximately half of the total weight [23], is rich in bioactive compounds including polyphenols, flavonoids, and tannins [24,25]. These secondary metabolites have been extensively studied for their antioxidant, antimicrobial, and antifungal properties [26,27,28,29]. Despite these properties, the bioactivity of pomegranate peel extracts towards insect species has been little investigated. Peel extracts exhibited significant insecticidal activity against the desert locust, *Schistocerca gregaria* (Forsk.) affecting adult performance and biochemical markers like transaminase activity [30]. Moreover, pomegranate extracts showed strong acaricidal effects against house dust mites [31] and effectively managed larvae and adult stages of the cotton leaf worm (*Spodoptera littoralis* (Boisduval)) [32]. Additionally, sublethal toxic effects were observed on the mite pest *Tetranychus urticae* Koch [33], as well as on the red palm weevil, *Rhynchophorus ferrugineus* (Olivier) [34]. Despite this expanding body of literature, to the best of our knowledge, limited research has focused specifically on stored-product insects. The granary weevil, *Sitophilus granarius* (Linnaeus) (Coleoptera: Curculionidae), is among the most damaging pests affecting stored cereal grains, particularly wheat. Its infestation results in substantial direct grain loss through consumption, and secondary damage due to mold development and increased moisture levels [35,36,37]. Currently, control strategies against *S. granarius* predominantly involve synthetic insecticides, mainly phosphine, organophosphates and pyrethroids, whose resistance issues and detrimental effects on human health and the environment reinforce the necessity for alternative sustainable approaches [38,39]. In this context, this study investigates the insecticidal and repellent activity of different pomegranate peel extracts against *S. granarius* adults. By leveraging the bioactive properties of this by-product, the research aims to contribute to the development of sustainable pest management solutions for stored cereals.

## 2. Materials and Methods

### 2.1. Insect Rearing

Adults of *S. granarius* (L.) were reared for six generations at the Department of Agriculture, Food, Natural Resources and Engineering of the University of Foggia. Insects were maintained on wheat grains (*Triticum aestivum* L.) in cylindrical glass containers (Ø 15 × 15 cm) closed with a metallic net (0.5 mm). The rearing was maintained in the dark at 25 ± 2 °C and 60 ± 5% relative humidity (r.h.). Newly emerged adults were collected daily and maintained under the same rearing conditions. Unsexed adult beetles, 3- to 4-weeks old, were used only once per experiment.

### 2.2. Plant Material

Pomegranate fruits (*Punica granatum* L.) (Ako cultivar) were collected in mid-September 2022 from organic farms located in Foggia (Italy) (GPS coordinates: 41.101740) at the full ripeness stage. The fertilization regimen included the application of 100 units of phosphorus at the start of vegetative growth, 150 units of nitrogen during the vegetative phase, and 150 units of potassium before fruit maturation.

### 2.3. Pomegranate Peels Extracts

Pomegranate peels (3.71 kg) were manually removed from different fruits. Peels were oven-dried at 27 ± 2 °C for 15 days until constant weight, reaching a total dry weight of 0.94 kg (corresponding to a 74.7% reduction in fresh weight). A low-temperature, forced-air drying process was adopted to preserve the chemical integrity and bioactivity of the plant material [40,41]. The dried peels were ground using an SM 100 mill (Retsch, Haan, Germany; 2001) and the resulting powder was sieved (0.5 mm mesh). Pomegranate peel material (100 g) was soaked in acetone, diethyl ether, or *n*-hexane solvents (300 mL) for 24 h at room temperature, kept in the dark, and gently hand-mixed four times for 5 min. The mixtures were then centrifuged (40,000 rpm, 10 min, T 10 °C), and the supernatant was filtered (Whatman No. 113) (Cytiva, Marlborough, MA, USA). Solvents were then removed under vacuum using a rotary evaporator (40 °C, 200 mbar) (Laborota 4000, Heidolph, Schwabach, Germany). Each extraction was repeated twice to ensure procedural consistency. The residues obtained from *n*-hexane, diethyl ether, and acetone extracts, respectively, of 1.4 ± 0.4, 4.2 ± 0.6, 40.8 ± 12.3 g/kg dry weight, were stored at −20 °C.

### 2.4. Contact Toxicity Bioassay

The contact toxicity of different pomegranate peel extracts against *S. granarius* adults was assessed through topical application [42,43]. The extracts were dissolved in their respective solvents (acetone, diethyl ether and *n*-hexane), and two-fold serial dilutions were prepared (150.00 to 18.75 μg/μL). A 0.5 μL aliquot of each dilution was directly applied onto the pronotum of *S. granarius* adults in a temporary state of immobility (thanatosis) (700 series, Microliter™, Hamilton Company, Reno, NV, USA). Each concentration was tested on three groups of 10 unsexed adults. Replicates were set up on the same day under identical laboratory conditions. Insects treated with solvent alone were used as controls. For each replicate, insects treated (*n* = 10) were transferred in a Petri dish and positioned within a metal ring (Ø 4.0 × 2.5 cm) covered with a metallic mesh (1 mm) to prevent escape. Five wheat kernels were provided to insects as food. The entire setup was kept in darkness at 26 ± 2 °C and 60 ± 5% relative humidity. Mortality rate was recorded after 24 and 48 h of extracts application. Individuals were considered dead when showing no movement even after gentle stimulation with a fine hair camel brush. The recorded mortality percentages were arcsine square-root transformed and submitted to one-way ANOVA analysis followed by Tukey’s HSD test for means comparison. Data normality (Shapiro–Wilk test) and variance homogeneity (Levene’s test) were assessed before analysis. The lethal doses required to achieve 50% and 90% mortality (${\mathrm{LD}}_{50}$ and ${\mathrm{LD}}_{90}$), alongside their upper and lower confidence limits, regression equations, and chi-square ($\chi^{2}$) values, were calculated through probit analysis [44]. Statistical analysis was performed using SPSS (Statistical Package for the Social Sciences) Version 23 for Windows (SPSS Inc., Chicago, IL, USA).

### 2.5. Ingestion Toxicity, Antifeedant, and Nutritional Activity

The effect of different pomegranate peel extracts on the feeding and nutritional indices of adult granary weevils was evaluated by the flour disk bioassay, following the method of Xie (1996) [45] with minor modifications [43]. Wheat flour (2.5 g) was gently mixed with distilled water (10 mL) using magnetic stirring. Flour disks were then obtained by depositing 100 μL aliquots of the mixture onto plastic Petri dishes which were subsequently left to dry overnight (26 ± 2 °C and 60 ± 5% r.h.) ensuring a consistent moisture content of the disks [46]. Flour disks were then treated with 5 μL of each extract solution, corresponding to 93.75, 187.50, 375.00, 750.00 μg/disk concentrations. Disks treated with solvent alone were used as control. To allow solvent evaporation, both treated and solvent-control disks were maintained at room temperature for 2 h before insect exposure. In a pre-weighed glass vial (Ø 2.5 × 4.0 cm), two treated flour disks were placed, and their weight was recorded. Subsequently, ten group-weighed weevil adults were introduced into each vial, which was then re-weighed and maintained in the darkness (26 ± 2 °C, 60 ± 5% r.h.) for three days. Post-experiment, the number of dead insects was recorded for each vial, and the weight of both the remaining flour disks and the surviving insects were separately determined. For each extract concentration and control, 5 replicates, each encompassing 10 insects, were set up on the same day. The following nutritional indices [47,48] were calculated for each replicate:

(1) $$\mathrm{Relative}\;\mathrm{Growth}\;\mathrm{Rate}\;\left(\mathrm{RGR}\right)=\frac{A-B}{B}\;{\mathrm{day}}^{-1}$$

(2) $$\mathrm{Relative}\;\mathrm{Consumption}\;\mathrm{Rate}\;\left(\mathrm{RCR}\right)=\frac{D}{B}\;{\mathrm{day}}^{-1}$$

(3) $$\mathrm{Efficiency}\;\mathrm{Conversion}\;\mathrm{of}\;\mathrm{Ingested}\;\mathrm{Food}\;\left(\mathrm{ECI}\right)=\frac{RGR}{RCR}\times 100$$

(4) $$\mathrm{Feeding}\;\mathrm{Deterrence}\;\mathrm{Index}\;\left(\mathrm{FDI}\right)=\frac{C-T}{C}\times 100$$

where A is the mean weight (mg) of live insects on the third day; B is the initial mean weight (mg) of insects; C is the consumption of control disks; D is the biomass ingested (mg) per number of living insects on the third day; and T indicates the consumption of treated disks. Data normality (Shapiro–Wilk test) and variance homogeneity (Levene’s test) were assessed before performing ANOVA, followed by post hoc comparisons using Tukey’s HSD test.

### 2.6. Repellency Bioassay

The repellent activity of pomegranate peel extracts was evaluated using the area preference redmethod [43,49]. A filter paper disc (Whatman No. 1, Ø 8.0 cm, area = 50.2 cm^2^) was divided in half. One half was treated with 500 μL of pomegranate peel extract solution using a micropipette, while the other half was treated with an equal volume of the solvent alone as a control. Both the treated and control halves were air-dried for approximately 10 min to allow complete evaporation of the solvent. The two halves were then joined with transparent adhesive tape, and the full disc was fixed to the bottom of a Petri dish (Ø 9.0 cm). Ten unsexed adult weevils were confined on each filter paper disc within a metal O-ring (Ø 8.0 × 4.0 cm) covered with a metallic mesh (1 mm) to prevent escape. The experiment was conducted in darkness at 26 ± 2 °C and 60 ± 5% r.h. Solutions of pomegranate peel extracts, prepared as described in Section 2.3, were tested at concentrations of 0.37, 0.75, 1.49, and 2.98 mg/cm^2^, respectively. For each extract concentration 4 replicates, each encompassing 10 insects, were set up on the same day. The number of weevils on the treated (Nt) and control (Nc) portions of the paper disc was recorded after 10 min, 30 min, 1 h, 2 h, and 24 h. The percent repellency (PR) of each extract was calculated using the following formula:

(5) $$\mathrm{Percent}\;\mathrm{Repellency}\;\left(\mathrm{PR}\right)\;\left(\%\right)=\frac{\left(N_{c}-N_{t}\right)}{\left(N_{c}+N_{t}\right)}\times 100$$

where $N_{c}$ is the number of insects present in the control half paper and $N_{t}$ the number of insects present in the treated one. Data normality and variance homogeneity were verified using the Shapiro–Wilk and Levene tests, respectively. PR values were subjected to repeated-measures ANOVA, with Greenhouse–Geisser correction applied when Mauchly’s test of sphericity was violated. Means at each exposure time were separated by Tukey’s HSD test (*p* < 0.05).

### 2.7. Gas Chromatography–Mass Spectrophotometry (GC-MS) Analysis

The crude acetone extract of pomegranate peels was diluted 1:100 with acetone and a 1 μL sample was injected in the gas chromatographic system. A 7890B gas chromatograph (Agilent Technologies, Santa Clara, CA, USA) with an Agilent 5977 mass selective detector (MSD) and equipped with a HP-5MS capillary column (30 m × 0.25 mm i.d., 0.5 μm film thickness, Agilent Technologies, Santa Clara, CA, USA) was used. The carrier gas was helium at a flow rate of 1.25 mL/min. The injection was made in the splitless mode, and the injector temperature was 250 °C. The oven temperature was initially held at 100 °C for 5 min then programmed to 280 °C at 6.0 °C/min with a final holding time of 40 min. Spectra were recorded in the electron impact mode (ionization energy, 70 eV) in the range of 35–550 amu at 2.9 scans/s. A solvent delay time of 5 min was used to avoid overloading the mass spectrometer with the solvent. The identification of compounds was achieved by comparing mass spectra with those of the data system library (NIST11). Moreover, a mixture of a continuous series of straight-chain hydrocarbons, C8–C20 (Alkane Standard Solution C8–C20, Sigma Aldrich, Milan, Italy) was injected into the HP-5MS column under the same conditions previously described for the acetone peels extract to calculate the linear retention indices (RIs), according to the method of van den Dool and Kratz (1963) [50]. A compound was considered identified when the spectral similarity exceeded 90% and the experimental RI corresponded to the RI reported in the literature (NIST Chemistry WebBook or scientific sources for the HP-5MS column) within ±10 units. Component relative percentages were calculated based on GC peak areas. Due to the known limitations of non-polar columns such as the HP-5MS in separating β-, γ-, and α-sitosterol isomers, the peak detected at RI 3203 was assigned to an isomeric mixture of sitosterols, as previously reported [51,52,53].

## 3. Results

### 3.1. Contact Bioassay

Mortalities of *S. granarius* adults 24 and 48 h after topical application of different pomegranate peel extracts are presented in Table 1, Table 2 and Table 3. Among all extracts, the strongest contact toxicity was shown by acetone and *n*-hexane extracts whereas the diethyl ether extract exhibited a low toxicity. After 24 h treatment, acetone and *n*-hexane extracts induced significantly higher mortalities than control (ANOVA: acetone, F = 25.6, df = 4, *p* < 0.001; *n*-hexane, F = 52.9, df = 4, *p* < 0.001) starting from the 37.5 μg/adult dose whereas no significant mortality (F = 0.50, df = 4, *p* = 0.737) was recorded for diethyl ether extract at any dose tested. The 24 h ${\mathrm{LD}}_{50}$ values calculated for the *n*-hexane and acetone extracts were 81.14 and 76.93 μg/adult, respectively. These values slightly decreased 48 h after treatment (Table 1, Table 2 and Table 3).

### 3.2. Ingestion Toxicity, Antifeedant, and Nutritional Activity

The ingestion toxicity, antifeedant, and nutritional effects of pomegranate peel extracts towards *S. granarius* adults are summarized in Table 4, Table 5 and Table 6. Overall, mortality was low across all treatments. The acetone extract induced a significant dose-dependent reduction in relative growth rate (RGR, F = 9.25; df = 4; *p* = 0.002), relative consumption rate (RCR, F = 9.05; df = 4; *p* = 0.002) and efficiency of conversion of ingested food (ECI, F = 14.94; df = 4; *p* < 0.01). In contrast, the *n*-hexane and diethyl ether extracts elicited no appreciable variation in these parameters, with values remaining statistically comparable to those of the corresponding controls (*p* > 0.05). The feeding deterrence index (FDI) showed a significant dose-dependent increase exclusively for the acetone extract, reaching the highest value of 75.13% at the highest concentration (F = 40.57; df = 4; *p* < 0.001) tested. In contrast, diethyl ether and *n*-hexane extracts exhibited positive but non-significant FDI values across all concentrations (F = 1.3–1.1, df = 4, *p* > 0.05).

### 3.3. Area Preference Bioassay

The repellent activity of pomegranate peel extracts against *S. granarius* adults was evaluated using filter paper disc bioassays. Among the extracts tested, the acetone extract was the most effective, particularly at the highest dose (2.98 mg, F = 6.24, df = 3, *p* < 0.05), achieving a PR of 80% after 10 min of exposure (Table 7). In contrast, the *n*-hexane and diethyl ether extracts exhibited markedly lower and less consistent repellent effects (Table 8 and Table 9). Statistical analysis confirmed a significant effect of dose (F = 15.76; *p* < 0.001) and time (F = 6.24; *p* < 0.001), with no significant dose × time interaction (*p* = 0.300) (Table 10) for acetone extract. The *n*-hexane extract showed a significant effect of dose (F = 5.05; *p* = 0.017), whereas the diethyl ether extract did not show any significant effect with dose (F = 1.37; *p* = 0.300), time (F = 0.30; *p* = 0.827), or their interaction (F = 1.57; *p* = 0.132) (Table 11 and Table 12).

### 3.4. Gas Chromatography–Mass Spectrophotometry (GC-MS) Analysis

The main components of the pomegranate peel acetone extract and their peak area (%) are presented in Table 13. The extract chemical profiling revealed the presence of 10 compounds corresponding to 95% of the total extract. Squalene (3.63%), 5-hydroxymethylfurfural (7.34%), α-tocopherol (9.82%), hexadecanoic acid (12.29%), 9,12-octadecadienoic acid (25.14%), and sitosterol (34.46%) along with 4 other minor compounds with relative peak areas ranging from 0.44 to 0.77% were identified.

## 4. Discussion

Among fruit by-products, pomegranate peels are particularly rich in bioactive compounds [54]. This study indicates that pomegranate peel extracts possess insecticidal potential against *S. granarius* adults, depending on the solvent used for extraction (acetone, *n*-hexane, and diethyl ether). Notably, in contact toxicity bioassays, the acetone and *n*-hexane extracts achieved the highest mortality rates (70–77%), contrasting with the very low toxicity observed for the diethyl ether extract (3%). Comparable results have been reported for extracts from other botanical matrices [55,56], underscoring the crucial role of solvent selection in optimizing the recovery of insecticidal bioactive compounds.

The contact toxicity of the acetone pomegranate peel extract against the granary weevil in this study (${\mathrm{LD}}_{50}=76.93$ μg/adult) was lower than that reported for the acetone extract from *Humulus lupulus* (L.) aerial parts (${\mathrm{LD}}_{50}=16.17$ μg/adult) [43], but higher than that of the acetone extract from *Sideritis trojana* (Bornm.) (${\mathrm{LD}}_{50}=136.60$ μg/adult) [57]. The contact toxicity of the *n*-hexane pomegranate peel extract (${\mathrm{LD}}_{50}=81.14$ μg/adult) was markedly lower than that of *Melia azedarach* (L.) (${\mathrm{LD}}_{50}=1.7$ μg/adult) [58] but comparable to that of *Dittrichia viscosa* (L.) (${\mathrm{LD}}_{50}=53.2$ μg/adult) [42].

Considering other stored-product insect pests, pomegranate peel ethanolic extracts have exhibited strong contact toxicity against *Tribolium castaneum* (Herbst) larvae and adults [59]. Similarly, El-Sayed and Said (2017) [60] reported strong contact insecticidal effects of a methanolic pomegranate peel extract against *Callosobruchus maculatus* (Fabricius) adults, with an ${\mathrm{LD}}_{50}$ of 1.22%. The contact toxicity observed in this study is therefore consistent with previous findings on other pest species. For instance, El Sakaan et al. (2024) [61] observed significant larval mortality in *S. littoralis* after treatment with ethanolic pomegranate peel extracts, while Jung (2015) [31] reported 100% mortality of house dust mites following exposure to pomegranate peel extracts prepared with the same solvent.

In ingestion bioassays, the acetone extract also significantly affected both nutritional and growth parameters of the granary weevil, in contrast to the *n*-hexane and diethyl ether extracts. Most notably, at the highest concentration tested (750 μg/disk), the acetone extract exhibited a strong feeding deterrence (75%). No antifeedant effect was found for extracts from *Scrophularia canina* (L.) or *D. viscosa* against the same pest [42,62]. Moreover, the high FDI value of the acetone extract contrasts with the low antifeedant effects observed for other plant-based extracts against stored-product insect pests. For instance, a methylene chloride extract of *Cinnamomum aromaticum* (Nees) induced only 35% feeding deterrence in *Sitophilus zeamais* (Motschulsky) adults and near-zero FDI values in *T. castaneum* larvae and adults [63]. Furthermore, Haridasan et al. (2017) [64] reported an FDI of about 66% for *Vitex negundo* (L.) leaf extracts towards *T. castaneum*. The antifeedant activity of the acetone pomegranate peel extract in this study also aligns with previous findings on *S. littoralis* larvae, which showed strong antifeeding responses to ethanol and methanol pomegranate peel extracts (92.96% ± 2.92 and 77.63% ± 3.78, respectively) [65].

Our contact repellency assays showed a dose-dependent effect of pomegranate peel extracts, with the acetone extract reaching up to 80% repellency at the highest concentration tested (2.98 mg/cm^2^). The *n*-hexane extract showed a more moderate repellency, not exceeding 50%. Similar repellent effects against *S. granarius* were also reported for extracts from *H. lupulus* [43] and *Prangos ferulacea* (Lindley) [66]. Interestingly, ethanolic pomegranate peel extracts have produced contrasting results in different species. Mohammad et al. (2012) [67] reported up to 100% contact repellency against *T. confusum* at the highest dose tested (10%), while Hamouda et al. (2014) [59] observed limited repellency (≤20%) against *T. castaneum* at a 2% concentration. Such discrepancies suggest that, beyond concentration, the susceptibility to *P. granatum* extracts may vary considerably among insect species.

Repellent materials can provide immediate protection to stored cereals by deterring insects before feeding or oviposition, thereby limiting damage and preventing population growth. Moreover, because repellents do not necessarily kill pests, they exert lower selection pressure, potentially reducing the likelihood of resistance development [68,69]. The strong repellency of the pomegranate peels acetone extract observed in this study could be used to complement integrated pest management strategies by combining repellent and attractive compounds, according to a push-pull approach, to manipulate insect behaviour and distribution. In addition, this extract could be applied to prevent insect infestation in storage facilities or used as a chemical barrier to protect packaged food products from insect invasion [70,71].

The GC–MS analysis of the acetone extract revealed sitosterol (isomeric mixture) as one of the major constituents, accounting for approximately 34.46% of the total extract. This phytosterol is widely distributed in plants, and its biological activity against several insect pests has been reported. For instance, β-sitosterol may interfere with insect cholesterol metabolism, reducing larval development and adult emergence, as documented in *Helicoverpa armigera* (Hübner) and *Dermestes maculatus* (Fabricius), where its accumulation due to inefficient metabolism leads to toxicity [72,73]. Beyond its physiological effects, β-sitosterol from petroleum ether extract of *Abutilon indicum* (L.) has demonstrated larvicidal activity against *Culex quinquefasciatus* (Say), a mosquito species of medical importance [74]. In addition to sitosterol, other compounds identified in the acetone extract, including squalene, 5-hydroxymethylfurfural (5-HMF), α-tocopherol, hexadecanoic acid, 9,12-octadecadienoic acid, and 2,4-di-tert-butylphenol, have also been reported to exhibit insecticidal activity [75,76,77,78,79,80,81,82].

In conclusion, pomegranate peel is a rich source of bioactive compounds with insecticidal, antifeedant, and repellent activities. However, further studies are required to clarify the role of individual constituents, assess sublethal effects, and validate efficacy under field conditions, as well as to extend evaluations to other insect pests and non-target organisms.

## Figures and Tables

**Table 1:** Contact toxicity of different concentrations of the *n*-hexane extract of pomegranate peel against adults of *S. granarius* after 24 and 48 h from topical application. For each exposure time, mean mortality values followed by the same letter are not significantly different at p≤0.05 (Tukey’s HSD test).

Dose (μg/Adult)	Exposure Time (h)	% Mortality (Mean ± S.E.)	Regression Equation	$\chi^{2}$	LD_50_ (95% CL, μg/Adult)	LD_90_ (95% CL, μg/Adult)
150		73.33 ± 3.33 a	y=−4.24−2.22x	0.487	81.14 (62.47–114.80)	306.37 (188.26–826.73)
75		43.33 ± 3.33 b				
37.5		26.67 ± 3.33 b				
18.75		6.67 ± 3.33 c				
0	24 h	0.00 ± 0.00 c				
F		52.9				
d.f.		4				
*p*		<0.001				
150		76.67 ± 3.33 a	y=−4.44−2.36x	0.315	75.68 (59.06–102.96)	264.08 (170.50–619.14)
75		46.67 ± 6.67 ab				
37.5		26.67 ± 3.33 b				
18.75		6.67 ± 3.33 c				
0	48 h	0.00 ± 0.00 c				
F		57.5				
d.f.		4				
*p*		<0.001				

**Table 2:** Contact toxicity of different concentrations of the acetone extract of pomegranate peel against adults of *S. granarius* after 24 and 48 h from topical application. For each exposure time, mean mortality values followed by the same letter are not significantly different at p≤0.05 (Tukey’s HSD test).

Dose (μg/Adult)	Exposure Time (h)	% Mortality (Mean ± S.E.)	Regression Equation	$\chi^{2}$	LD_50_ (95% CL, μg/Adult)	LD_90_ (95% CL, μg/Adult)
150		76.67 ± 8.82 a	y=−4.84−2.57x	0.938	76.93 (60.95–102.14)	231.76 (161.52–243.08)
75		46.67 ± 12.02 ab				
37.5		26.67 ± 8.82 b				
18.75		3.33 ± 3.33 c				
0	24 h	0.00 ± 0.00 c				
F		25.6				
d.f.		4				
*p*		<0.001				
150		83.33 ± 6.67 a	y=−4.74−2.61x	0.362	65.31 (51.87–84.35)	202.45 (140.44–392.09)
75		53.33 ± 8.82 ab				
37.5		30.00 ± 5.77 bc				
18.75		6.67 ± 3.33 cd				
0	48 h	0.00 ± 0.00 d				
F		34.7				
d.f.		4				
*p*		<0.001				

**Table 3:** Contact toxicity of different concentrations of the diethyl ether extract of pomegranate peel against adults of *S. granarius* after 24 and 48 h from topical application. For each exposure time, mean mortality values followed by the same letter are not significantly different at p≤0.05 (Tukey’s HSD test).

Dose (μg/Adult)	Exposure Time (h)	% Mortality (Mean ± S.E.)	Regression Equation	$\chi^{2}$	LD_50_ (95% CL, μg/Adult)	LD_90_ (95% CL, μg/Adult)
150		3.33 ± 3.33 a	y=−3.08−0.63x	0.831	83,757.38	9,332,208.94
75		3.33 ± 3.33 a				
37.5		3.33 ± 3.33 a				
18.75		0.00 ± 0.00 a				
0	24 h	0.00 ± 0.00 a				
F		0.50				
d.f.		4				
*p*		0.737				
150		10.00 ± 5.77 a	y=−3.86−1.25x	1.185	1212.40	12,772.71
75		10.00 ± 0.00 a				
37.5		3.33 ± 3.33 a				
18.75		0.00 ± 0.00 a				
0	48 h	0.00 ± 0.00 a				
F		3.63				
d.f.		4				
*p*		0.045				

**Table 4:** Nutritional indices, mortality and food deterrence of *S. granarius* adults exposed to different concentrations of the *n*-hexane extract of pomegranate peel. Means in the same column with the same letter are not significantly different at the 0.05 level determined by Tukey’s HSD test.

Concentration (μg/Disk)	Mortality (%)	RGR (mg/mg/Day) (Mean ± S.E.)	RCR (mg/mg/Day) (Mean ± S.E.)	ECI (%) (Mean ± S.E.)	FDI (%) (Mean ± S.E.)
750.00	26.00 a	−0.018±0.009 a	0.089±0.034 a	−19.709±11.770 a	32.444±10.691 a
375.00	20.00 ab	−0.016±0.012 a	0.091±0.006 a	−19.806±13.195 a	34.467±1.931 a
187.50	14.00 ab	−0.011±0.013 a	0.091±0.014 a	−17.215±13.836 a	30.791±10.090 a
93.75	2.00 b	0.007±0.010 a	0.113±0.018 a	−12.119±11.842 a	10.778±14.826 a
Control	0.00 b	0.010±0.005 a	0.125±0.014 a	6.504±4.353 a	

**Table 5:** Nutritional indices, mortality and food deterrence of *S. granarius* adults exposed to different concentrations of the acetone extract of pomegranate peel. Means in the same column with the same letter are not significantly different at the 0.05 level determined by Tukey’s HSD test.

Concentration (μg/Disk)	Mortality (%)	RGR (mg/mg/Day) (Mean ± S.E.)	RCR (mg/mg/Day) (Mean ± S.E.)	ECI (%) (Mean ± S.E.)	FDI (%) (Mean ± S.E.)
750.00	20.00 a	−0.063±0.006 a	0.071±0.012 a	−97.566±15.177 a	75.131±3.467 a
375.00	14.00 ab	−0.066±0.005 a	0.088±0.009 a	−79.050±11.455 a	66.728±2.424 ab
187.50	10.00 ab	−0.060±0.002 a	0.112±0.010 ab	−56.152±7.901 ab	56.056±4.806 b
93.75	6.00 ab	−0.057±0.115 a	0.136±0.013 b	−31.950±9.887 bc	13.298±5.779 c
Control	0.00 b	0.010±0.006 b	0.144±0.006 b	4.886±4.353 c	

**Table 6:** Nutritional indices, mortality and food deterrence of *S. granarius* adults exposed to different concentrations of the diethyl ether extract of pomegranate peel. Means in the same column with the same letter are not significantly different at the 0.05 level determined by Tukey’s HSD test.

Concentration (μg/Disk)	Mortality (%)	RGR (mg/mg/Day) (Mean ± S.E.)	RCR (mg/mg/Day) (Mean ± S.E.)	ECI (%) (Mean ± S.E.)	FDI (%) (Mean ± S.E.)
750.00	16.00 a	−0.002±0.009 a	0.094±0.018 a	−17.864±23.316 a	32.333±14.221 a
375.00	22.00 a	−0.005±0.010 a	0.101±0.017 a	−9.635±7.827 a	31.750±15.803 a
187.50	12.00 ab	−0.004±0.006 a	0.095±0.008 a	−3.140±7.563 a	33.800±3.878 a
93.75	0.00 b	0.002±0.007 a	0.128±0.015 a	−0.096±5.510 a	6.000±9.176 a
Control	0.00 b	0.008±0.010 a	0.132±0.008 a	4.936±6.971 a	

**Table 7:** Percent repellency (PR) (±S.E.) of different concentrations of the *n*-hexane extract of pomegranate fruit peels against *S. granarius* adults in filter paper disc bioassays after different exposure times. Values in the same column followed by different letters are significantly different at p<0.05 (Tukey HSD test).

Dose (mg/cm^2^)	Exposure Time				
	**10 min**	**30 min**	**60 min**	**120 min**	**24 h**
2.98	45.00±9.57 a	45.00±9.58 a	25.82±12.91 a	50.00±12.91 a	30.00±12.91 a
1.49	25.00±9.57 ab	25.00±15.00 ab	20.00±8.16 a	20.00±21.60 a	50.00±12.91 a
0.75	−25.00±9.57 bc	−25.00±9.57 bc	5.00±20.61 a	5.00±20.61 a	5.00±20.61 a
0.37	−20.00±14.14 c	−15.00±5.77 b	10.00±20.82 a	5.00±5.00 a	5.00±17.08 a
F	9.9	8.7	1.5	1.7	1.8
d.f.	3	3	3	3	3
*p*	0.000	0.000	0.000	0.000	0.000

**Table 8:** Percent repellency (PR) (±S.E.) of different concentrations of the acetone extract of pomegranate fruit peel against *S. granarius* adults in filter paper disc bioassays after different exposure times. Values in the same column followed by different letters are significantly different at p<0.05 (Tukey HSD test).

Dose (mg/cm^2^)	Exposure Time				
	**10 min**	**30 min**	**60 min**	**120 min**	**24 h**
2.98	80.00±8.16 a	55.00±9.58 a	55.00±5.00 a	30.00±12.91 a	30.00±12.91 a
1.49	60.00±8.16 ab	40.00±8.16 a	40.00±8.16 ab	20.00±8.16 a	20.00±8.16 a
0.75	30.00±10.00 bc	25.00±15.00 a	−5.00±20.61 b	−10.00±12.91 a	−10.00±12.91 a
0.37	5.00±12.58 c	−30.00±5.77 b	−5.00±12.58 b	−10.00±12.91 a	−10.00±12.91 a
F	11.13	13.20	5.70	3.00	3.00
d.f.	3	3	3	3	3
*p*	0.0009	0.0004	0.0118	0.0728	0.0728

**Table 9:** Percent repellency (PR) (±S.E.) of different concentrations of the diethyl ether extract of pomegranate fruit peels against *S. granarius* adults in filter paper disc bioassays after different exposure times. Values in the same column followed by different letters are significantly different at p<0.05 (Tukey HSD test).

Dose (mg/cm^2^)	Exposure Time				
	**10 min**	**30 min**	**60 min**	**120 min**	**24 h**
2.98	10.00±20.82 a	−5.00±15.00 ab	10.00±20.82 a	10.00±17.32 a	15.00±12.58 a
1.49	−25.00±15.00 bc	10.00±5.77 a	−25.00±17.08 a	−25.00±17.08 a	15.00±15.00 a
0.75	15.00±12.58 bc	−10.00±12.90 ab	5.00±17.08 a	5.00±17.08 a	10.00±17.32 a
0.37	−20.00±16.33 c	−45.00±15.00 b	−15.00±15.00 a	−15.00±17.32 a	−15.00±20.61 a
F	1.6	4.8	0.9	1.1	0.7
d.f.	3	3	3	3	3
*p*	0.000	0.000	0.000	0.000	1.000

**Table 10:** Repeated measures analysis of variance between subjects effects for the repellent activity of the acetone extract of pomegranate fruit peels against *S. granarius* adults in filter paper disc bioassays at the doses of 2.98, 1.49, 0.75, and 0.37 mg/cm^2^ after 10, 30, 60, 120 min and 24 h exposure, respectively.

Source	df	Mean Square	F-Value	*p*-Value
Dose	3	17,258.333	15.761	<0.001
Error	12	1095		
Exposure time	4	2542.500	6.239	<0.001
Error	12	311.667		
Dose × Exposure time	12	495.833	1.217	0.300
Error	48	407.500		

**Table 11:** Repeated measures analysis of variance between subjects effects for the repellent activity of the *n*-hexane extract of pomegranate fruit peel against *S. granarius* adults in filter paper disc bioassays at the doses of 2.98, 1.49, 0.75, and 0.37 mg/cm^2^ after 10, 30, 60, 120 min and 24 h exposure, respectively.

Source	df	Mean Square	F-Value	*p*-Value
Dose	3	9773.333	5.046	0.017
Error	12	1936.667		
Exposure time	4	607.500	0.948	0.445
Error	12	1305		
Dose × Exposure time	12	527.500	0.823	0.626
Error	48	640.833		

**Table 12:** Repeated measures analysis of variance between subjects effects for the repellent activity of the diethyl ether extract of pomegranate fruit peels against *S. granarius* adults in filter paper disc bioassays at the doses of 2.98, 1.49, 0.75, and 0.37 mg/cm^2^ after 10, 30, 60, 120 min and 24 h exposure, respectively.

Source	df	Mean Square	F-Value	*p*-Value
Dose	3	3885	1.366	0.300
Error	12	2845		
Exposure time	4	129.167	0.298	0.827
Error	12	434.167		
Dose × Exposure time	12	739.167	1.573	0.132
Error	48	470		

**Table 13:** Chemical composition of acetone pomegranate peel extract.

Peak No.	Compound	^1^ R.T. (min)	^2^ RI_Exp_	^3^ RI_Lit_	Area (%)
	Hydrocarbons and Terpenes				
2	Tetradecene	10.92	1423	1425	0.63
3	trans-α-Bergamotene	11.94	1586	1584	0.44
5	(3E)-Elisocene	15.30	2022	2020	0.51
Total Hydrocarbons and Terpenes					1.58
	Lipids				
6	Hexadecanoic acid	22.11	1966	1964	12.29
7	9,12-Octadecadienoic acid	24.84	2089	2086	25.14
8	Squalene	33.68	2818	2820	3.63
9	α-Tocopherol	37.49	3132	3130	9.82
10	Sitosterols (isomeric mixture)	40.72	3200	3203	34.46
Total Lipids					85.34
	Others				
1	5-Hydroxymethylfurfural	7.38	1235	1233	7.34
4	2,4-Di-tert-butylphenol	13.74	1602	1605	0.77
Total Others					8.11

^1^ R.T. = retention time in minutes; ^2^ RI_Exp_ = Determined linear retention index against a mixture of *n*-alkanes (C_5_–C_40_) on HP-5MS column; ^3^ RI_Lit_ = Linear retention index from literature.

## Data Availability

The original contributions presented in this study are included in the article. Further inquiries can be directed to the corresponding author.
